# Prevalence of early initiation of breastfeeding and determinants of delayed initiation of breastfeeding: secondary analysis of the WHO Global Survey

**DOI:** 10.1038/srep44868

**Published:** 2017-03-21

**Authors:** Kenzo Takahashi, Togoobaatar Ganchimeg, Erika Ota, Joshua P. Vogel, João Paulo Souza, Malinee Laopaiboon, Cynthia Pileggi Castro, Kapila Jayaratne, Eduardo Ortiz-Panozo, Pisake Lumbiganon, Rintaro Mori

**Affiliations:** 1 Teikyo University Graduate School of Public Health, 2-11-1 Kaga, Itabashi-ku, Tokyo 173-8605, Japan; 2 Faculty of Medicine, University of Tsukuba, 1-1-1 Tennodai, Tsukuba, Ibaraki 305-8575, Japan; 3 Department of Health Policy, National Center for Child Health and Development, 2-10-1 Okura, Setagaya-ku, Tokyo 157-8535, Japan; 4 Department of Global Health Nursing, St. Luke’s International University, 10-1 Akashi-cho, Chuo-ku, Tokyo 104-0044, Japan; 5 UNDP/UNFPA/UNICEF/WHO/World Bank Special Programme of Research, Development and Research Training in Human Reproduction (HRP), Department of Reproductive Health and Research, World Health Organization, Avenue Appia 20, Geneva, Switzerland; 6 Faculty of Public Health, Department of Biostatistics & Demography, Khon Kaen University, 123 Moo 16 Mittapap Rd., Nai-Muang, Muang District, Khon Kaen 40002, Thailand; 7 Department of Pediatrics, Ribeirao Preto Medical School, University of São Paulo, Av. Bandeirantes, 3900, Monte Alegre, Brazil; 8 Family Health Bureau, Ministry of Health, 231 De Saram Place, Colombo 10, Sri Lanka; 9 National Institute of Public Health, Center for Population Health Research, Av. Universidad No. 655 Colonia Santa María Ahuacatitlán, Cerrada Los Pinos y Caminera C.P. 62100, Cuernavaca, México; 10 Department of Obstetrics & Gynaecology, Faculty of Medicine, Khon Kaen University, 123 Moo 16 Mittapap Rd., Nai-Muang, Muang District, Khon Kaen 40002, Thailand

## Abstract

Early initiation of breastfeeding (EIBF) within 1 hour of birth can decrease neonatal death. However, the prevalence of EIBF is approximately 50% in many developing countries, and data remains unavailable for some countries. We conducted a secondary analysis using the WHO Global Survey on Maternal and Perinatal Health to identify factors hampering EIBF. We described the coverage of EIBF among 373 health facilities for singleton neonates for whom breastfeeding was initiated after birth. Maternal and facility characteristics of EIBF were compared to those of breastfeeding >1 hour after birth, and multiple logistic regression analysis was performed. In total, 244,569 singleton live births without severe adverse outcomes were analysed. The EIBF prevalence varied widely among countries and ranged from 17.7% to 98.4% (average, 57.6%). There was less intra-country variation for BFI <24 hours. After adjustment, EIBF was significantly lower among women with complications during pregnancy and caesarean delivery. Globally, EIBF varied considerably across countries. Maternal complications during pregnancy, caesarean delivery and absence of postnatal/neonatal care guidelines at hospitals may affect EIBF. Our findings suggest that to better promote EIBF, special support for breastfeeding promotion is needed for women with complications during pregnancy and those who deliver by caesarean section.

Despite a significant reduction in child mortality from 12.7 million in 1990 to 5.9 million in 2015, neonatal mortality has been decreasing more slowly and constitutes a larger proportion of under-5 mortality^1^. Globally, neonatal mortality represented approximately 45% of under-5 deaths in 2015^2^. The World Health Organization (WHO) has recommended a package of interventions including breastfeeding to reduce neonatal mortality^3^. Breastfeeding is a unique, valuable feeding practice in infancy that is associated with lower neonatal mortality and which alleviates inequities in child mortality and prevents morbidities such as diarrhoea, pneumonia, neonatal sepsis and may reduce obesity and diabetes later in life^4^,^5^,^6^,^7^. An estimated 11.6% of infant deaths and 21.9 million disability-adjusted years could be prevented by large-scale breastfeeding promotion programmes^8^. The global breastfeeding recommendations are to place all newborns in skin-to-skin contact with their mothers immediately after birth, to support the initiation of breastfeeding (BFI) within 1 hour after birth (defined as early initiation of breast feeding or EIBF) and to exclusively breastfeed the child until 6 months of age^7^,^9^.

EIBF stimulates breast milk production, produces antibody protection for the newborn and reduces postpartum maternal haemorrhage, and its practice determines the successful establishment and longer duration of breastfeeding^10^,^11^,^12^. Several studies have shown that EIBF is associated with a lower risk of neonatal mortality^13^,^14^,^15^,^16^.

Despite the known health benefits of EIBF, in many countries, a considerable proportion of newborns are not breastfed within 1 hour after birth in accordance with the WHO recommendation. The prevalence of EIBF ranges from 14% to 95% with an average of 64% in 128 countries, and one-half of these countries have a prevalence of less than 50%^17^. Furthermore, existing studies were conducted in a single country or focused on individual factors only^10^. A recent systematic review of the literature on EIBF in South Asia, which included 25 studies from 7 countries, revealed that EIBF is predominately associated with socio-economic, health-related and individual factors, and it highlighted the limited evidence on the health care system in relation to EIBF^18^.

To address these gaps and promote EIBF, we need to acquire both data on EIBF coverage and a better understanding of the factors associated with delayed breastfeeding, for which little is known, especially in low- and middle income countries.

In this analysis, we aimed to determine the coverage of EIBF and individual and health facility factors associated with delayed EIBF taking advantage of the WHO Global Survey on Maternal and Perinatal Health (WHO GS) that was implemented concurrently in health facilities across 24 countries using a standardised questionnaire.

## Methods

We conducted a secondary data analysis of the WHO GS dataset. The methodological details of this survey were described previously^19^,^20^. The survey was implemented in 373 health facilities of 24 countries using a stratified multistage cluster sampling design. The countries included in the survey were from three continents: Africa (Algeria, Angola, Democratic Republic of Congo, Niger, Nigeria, Kenya and Uganda), Latin America (Argentina, Brazil, Cuba, Ecuador, Mexico, Nicaragua, Paraguay and Peru) and Asia (Cambodia, China, India, Japan, Nepal, Philippines, Sri Lanka, Thailand and Vietnam). For each country, two provinces were randomly selected with probability proportional to population size and were included in the sample along with the capital city of the country. From each of these selected areas, up to seven health facilities that had at least 1,000 deliveries per year and the capability to perform caesarean section were randomly selected with probability proportional to the number of births per year. The survey was conducted between 2004 and 2005 in Africa and Latin America and between 2007 and 2008 in Asia.

Trained medical staffs extracted data from patient records for all women who were admitted for delivery at the participating facilities during a 2- to 3-month period. Individual data were collected at hospital discharge, 8 days postpartum or at maternal death, whichever occurred first, and included demographics and reproductive characteristics, medical conditions during pregnancy, mode of delivery, birth outcomes, maternal and perinatal complications, received interventions and time of BFI.

In addition to individual data, facility information was collected on a standard form by the hospital coordinators in consultation with the facility director or the head of obstetrics, as described previously^20^. Data encompassed maternal and perinatal care characteristics, including the availability of laboratory tests, anaesthesiology resources, intrapartum care services, delivery and care of the newborn infant, and the availability of basic emergency medical and obstetric care facilities, intensive care units (ICUs) and human training resources.

### Study population

**Figure 1:**
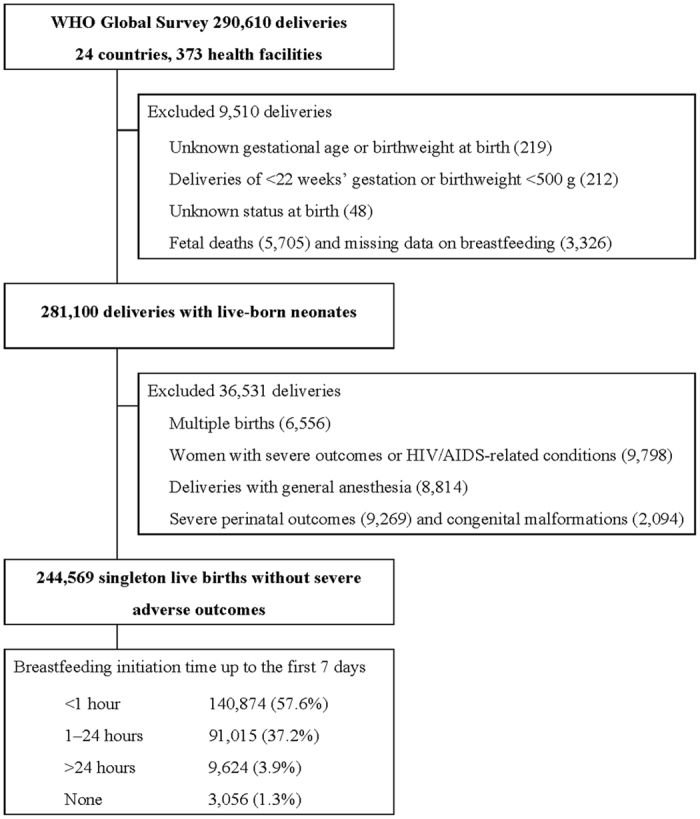
Flow chart of the study population.

In the WHO GS dataset, individual data were available for all mothers and neonates, including first-born neonates in the case of multiple births. We initially selected all live births excluding deliveries at <22 weeks’ gestational age, birth weight <500 g, stillbirths and deliveries with missing data on BFI. The study population was restricted to neonates and mothers considered able to initiate breastfeeding and excluded those with multiple births and deliveries with certain maternal and perinatal complications, including congenital malformation, neonatal near-miss cases^21^ at gestational age <33 weeks, birth weight <1750 g or Apgar score at 5 minutes <7, women with severe maternal outcomes^22^ defined as the presence of any of the following conditions: eclampsia, blood transfusion, hysterectomy, admission to ICU and maternal death, women with any conditions suggesting HIV/AIDS and deliveries with general anaesthesia (Fig. 1).

### Variables

EIBF was the main outcome (variable of interest of this analysis) of this study, and this outcome was designated as ‘Yes’ and all others were designated as ‘No’. Additionally, to describe the time to BFI in the participating facilities and countries, we used the following four categories: EIBF (<1 hour), BFI within 1–24 hours, BFI after the first day (>24 hours) and breastfeeding not initiated by the time of discharge or by 7 days after birth.

The following maternal and obstetric characteristics were considered as factors associated with EIBF: maternal age, marital status, education, parity, number of antenatal care visits, mode of delivery, gestational age at delivery and complications during pregnancy (defined as the presence of any of the following: pregnancy-induced hypertension, chronic hypertension, cardiac/renal diseases, chronic respiratory conditions, diabetes, severe anaemia [haemoglobin <7 g/dl], pyelonephritis or urinary tract infections and other health conditions). Also, health facility characteristics such as location, ownership, capacity and availability of postnatal and/or neonatal care were examined. Facility capacity was used to reflect the facilities’ medical and obstetric care service level and summarise the services available in each facility. Total scores were determined (range: 3–63) and categorised into three groups: low (<51), medium (51–60) and high (>61)^23^.

### Statistical analysis

We analysed the occurrence of BFI among 373 health facilities in 24 countries in singleton neonates who could breastfeed soon after birth. The prevalence of EIBF and BFI within 1–24 hours after birth was examined among participating health facilities. Maternal and facility characteristics of EIBF were compared to those of BFI at >1 hour after birth, and multiple logistic regression analysis was performed to determine odds ratios (OR) and adjusted OR (AOR). All missing variables were excluded from the analysis of association estimates. Analysis was conducted to account for the complexity of the study design in which health facilities were considered as sampling units and countries as strata. Statistical analysis was conducted using Stata/MP version 13·0 (Stata Corp LP, College Station, TX), and a P value of <0·05 was considered to indicate statistical significance.

### Role of the funding source

The funders had no role in the study design, data collection and analysis, decision to publish or preparation of this manuscript.

### Ethical approval

The WHO GS was approved by the WHO Ethical Review Committee and the relevant ethical clearance bodies in the participating countries and facilities. Informed consent was formally waived by the WHO Ethical Review committee. Thus, written consent from individual women was not needed because there was no contact between the data collectors (who extracted routine medical records data) and the individual women.

## Results

**Figure 2:**
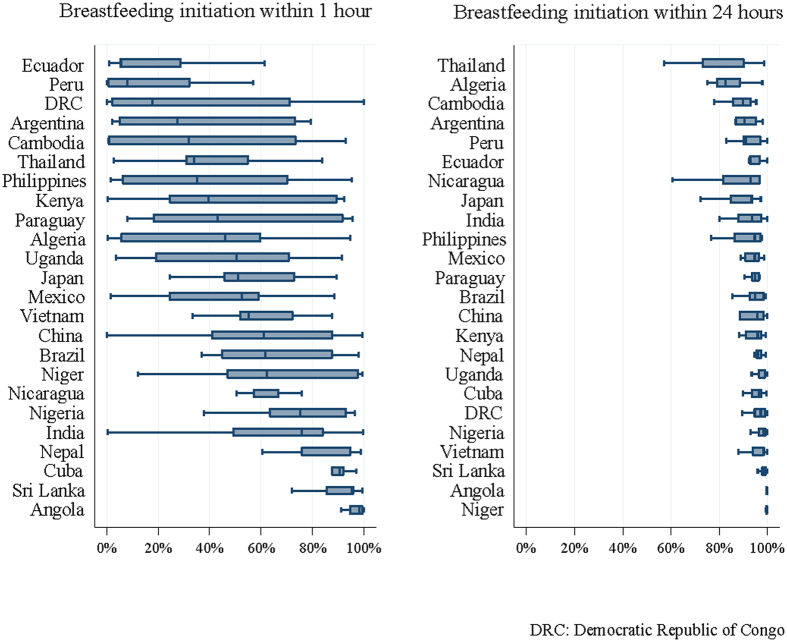
Variation in the initiation of breastfeeding practices at participating health facilities by country (median ranges and 25^th^ and 75^th^ percentiles).

Data from 290,610 deliveries were collected from 373 health facilities in the 24 countries in the WHO GS. BFI data were available for 281,100 deliveries with liveborn neonates. Of these, 244,569 singleton live births were included in this analysis after applying the exclusion criteria (Fig. 1). BFI practice in the study population is shown in Table 1. Overall, breastfeeding was initiated for 57.6% and 37.2% of neonates within the first hour after birth and from 1–24 hours after birth, respectively. The proportion of EIBF among all live births ranged from 17.7% to 98.4% with the lowest percentages found in Peru (17.7%), Ecuador (20.1%) and the Philippines (39.9%) and the highest in Angola (98.4%), Cuba (89.2%) and Sri Lanka (88.5%). We observed wide variation in EIBF both within and between countries. However, the variation in BFI within the first 24 hours after birth was narrower than that for EIBF (Fig. 2).

Characteristics of the study population are shown in Table 2. The majority of mothers were aged 20–34 years (76.9%), married (86.9%), had made at least four antenatal care visits (67.5%) and delivered vaginally (78.3%) at 37–41 weeks’ gestation (89.7%). Furthermore, most deliveries took place at a public facility in an urban setting.

Table 3 shows ORs and AORs for the estimated effects of individual and facility characteristics for EIBF. The crude OR for EIBF was significantly lower among women aged >35 years (OR 0.85; 95% CI 0.73–0.96) and having a secondary (OR 0.83; 95% CI 0.70–0.99) or higher education (OR 0.76; 95% CI 0.59–0.97) compared to women aged 20–34 years and having a primary or lower education, respectively. Odds of EIBF were significantly lower in Latin America (OR 0.63; 95% CI 0.41–0.99) than in Africa. However, after adjustment for potential confounders, these lower odds were no longer significant.

EIBF was significantly lower among women who had complications during pregnancy (AOR 0.76; 95% CI 0.65–0.88) and those who delivered by caesarean section (AOR 0.28; 95% CI 0.22–0.37). Deliveries at facilities with available postnatal and/or neonatal guidelines/protocols were more likely to be associated with EIBF (AOR 2.05; 95% CI 1.07–3.92) than those at facilities with no guidelines.

## Discussion

In this study, we investigated EIBF among women and their singleton neonates without maternal and perinatal severe adverse outcomes. We found that maternal complications during pregnancy, caesarean delivery (CD) and absence of postnatal/neonatal care guidelines at hospitals were negatively associated with EIBF. Maternal socio-demographic characteristics were not found to be factors associated with EIBF.

As we noted in the Introduction, there is a research gap relating to EIBF promotion, especially considering the substantial evidence indicating that EIBF is suitable for practical intervention. Research in developed countries suggests that health education and peer support interventions^24^, the duration of labour^25^, having a professional occupation^26^ and maternal overweight^27^ are associated with the initiation of breastfeeding. However, the duration of labour or having a professional occupation are not modifiable factors, and the generalisability and relevance of these findings to low- and middle- income countries is questionable. Comparatively little evidence is available relating to the determinants of BF in low- and middle-income countries. In a cohort study of exclusive breastfeeding practices in eight countries, Patil *et al*. found that primiparity, the provisioning of prelacteal foods and the withholding of colostrum are all associated with delay in the initiation of breastfeeding^28^. On the basis of these facts and keeping our findings in mind, we discuss feasible policy implications for the promotion of EIBF such as special support for mothers with CD and maternal complications.

Overall 57.6% of mothers in our study initiated breastfeeding within 1 hour after birth. This result was similar to recent data on breastfeeding indicators from 153 countries that showed the prevalence of EIBF to range from 30% to 60% in low-, middle- and high-income countries^7^.

The proportions of EIBF practiced at health facilities varied widely within (0–100%) and between (17.7–98.4%) the participating countries of the WHO GS. These were variations likely due to factors such as cultural and economic characteristics^29^, prelacteal feeding^28^ and violation of the 1981 International Code of Marketing for Breastmilk Substitutes^30^ and health institutional practices and policies^31^.

Our results showed that CD was one of the factors contributing to the delay in the initiation of breastfeeding, which is consistent with previous studies^32^,^33^. A recent systematic review provided evidence that CD is significantly negatively associated with EIBF. The authors suggested that maternal and foetal indications for CD and postoperative care disrupt bonding and mother-newborn interaction and delay BFI. The study also found greater risk of delayed BFI in elective, pre-labour CD and suggested a possible relation between maternal preference for CD and the decision not to breastfeed^34^. However, another meta-analysis suggested that CD is not a risk factor of EIBF in the presence of adequate support, but the details were not discussed^31^. Considering the increasing rates of CD globally, it is crucial to encourage and support EIBF in all women regardless of the mode of delivery and to inform all prospective mothers and health staff of the negative effects of CD on breastfeeding and the well-being of the newborn.

In this study, complications during pregnancy were associated with delayed EIBF, and it is possible that complications are more likely to cause intrapartum and early postpartum morbidities requiring immediate interventions for mothers or neonates that delay the mother-newborn interaction. Prevention of pregnancy complications and special support for mothers with complications, which are linked with postnatal care after hospital discharge, are essential to promoting EIBF. Although WHO recommends only 24 hours of care for mothers and neonates discharged from hospitals^35^, reflecting the positive evidence of postnatal care given in the communities in low- and middle-income countries^36^, the implementation of Birth Preparedness and Complication Readiness (BPCR) should be considered to reduce maternal and neonatal health risks^37^,^38^. A systematic review of BPCR by Soubeiga *et al*. found that the BPCR interventions associated with increased use of early newborn care included EIBF in low-resource settings^37^. We expect that the promotion of BPCR in the community works as a function of postnatal care. A feasible approach would be to integrate EIBF into primary health care (PHC) activities because PHC activities may entrench EIBF behaviours^1^,^39^,^40^. Strong evidence shows that lay health workers, who are key actors in PHC, aim to achieve BFI and a reduction in neonatal mortality through interventions by providing special support to women with caesarean section and complications during pregnancy^41^,^42^. We expect that a PHC programme that makes greater use of women’s groups and community health workers will be of benefit to low-income populations^43^,^44^,^45^.

The presence of guidelines for postnatal or neonatal care at health facilities was associated with a two times higher rate of EIBF, whereas maternal socio-demographic characteristics such as age, education and parity were not. Although an association with the presence of guidelines was found, it may be due to underlying associations between the availability of guidelines and the overall quality of care. The pursuit of contents of care may be an interesting pathway of investigation, and it can be our future challenge.

With further exploration of the data, we found that 95% of mothers initiated breastfeeding within the 1^st^ day of birth. Although some evidence exists of the association between the workloads of health care staff and breastfeeding promotion, considering that the workload of nurses affects patient safety^46^,^47^, we assume that another contributing factor to delayed EIBF is possibly related to health facility practices or to high workload and a shortage of human resources to support and promote EIBF in women immediately after birth because the WHO GS was conducted in relatively larger health facilities from mainly low- and middle-income countries. Our assumption can be supported by the past research, which implies that heavy workload may cause possible failure to provide appropriate advice for deliveries^48^. However, it is difficult to prove our assumption based on the available data. Thus, it may be interesting to explore the real situations of facility practices and staff workloads using the workload indicators for staffing need developed by WHO^49^, especially in the facilities studied in this survey. There are findings supportive of our assumption of greater hospital influence on EIBF practices regardless of the study setting or outcome duration^50^,^51^,^52^. Several studies found higher proportions of EIBF and longer durations of breastfeeding among mothers delivered at Baby Friendly Hospital Initiative (BFHI)-accredited hospitals and a positive association between the number of BFHI steps in place and breastfeeding outcomes^52^,^53^.

In spite of the existence of WHO and UNICEF recommendations and the BFHI since 1989, recently, only about half of newborns have begun breastfeeding within 1 hour of birth globally^7^. Therefore, expansion, monitoring and reaccreditation of BFHI, and adherence of clinical care standards would increase breastfeeding outcomes including EIBF, exclusive breastfeeding and a longer duration of breastfeeding.

A key strength of this study is the uniqueness of the dataset. The study data were collected concurrently during the same time period using standardised record forms right before discharge of the women from hospital at a large number of facilities in multiple countries. This allowed a comparison of EIBF practices, which is one of the steps of the BFHI, between countries and facilities.

This study also has several limitations. Because it is a cross-sectional study, we could not examine the causal relation of exposure and outcome variables^22^. Our study findings are not necessarily generalisable to community settings or smaller facilities. In addition, not all participating countries had high coverage of institutional delivery. In the WHO-GS data, information on cultural factors, maternal knowledge and intention to breastfeed, prelacteal feeding and the availability of facility-level breastfeeding policies and their compliance was not available.

Finally, BFI timing data was collected in four categories (<1 hour, 1–24 hours, >24 hours and breastfeeding not initiated before hospital discharge or by the 7^th^ day after birth) instead of at exact hours of initiation, which may have an important effect on showing how close to or far facilities are from improving EIBF coverage. However, despite these limitations, the WHO GS is a large, multi-country study that collected data in a standardised fashion.

In conclusion, our findings suggest that CD, maternal complications during pregnancy and the absence of postnatal/neonatal care guidelines were negatively associated with the rate of EIBF. To better promote EIBF, special support for the promotion of breastfeeding is needed for women with complications during pregnancy and those who deliver by caesarean section.

## Additional Information

**How to cite this article**: Takahashi, K. *et al*. Prevalence of early initiation of breastfeeding and determinants of delayed initiation of breastfeeding: secondary analysis of the WHO Global Survey. *Sci. Rep.* **7**, 44868; doi: 10.1038/srep44868 (2017).

**Publisher's note:** Springer Nature remains neutral with regard to jurisdictional claims in published maps and institutional affiliations.

## Figures and Tables

**Table 1:** Breastfeeding initiation practice by hospital discharge in participating countries.

Region	Country	Health facilities	Live births	Breastfeeding initiation n, %							
				≤1 hour		>1 to ≤24 hours		>24 hours		None	
Africa	Algeria	18	12,391	5,532	44.7	6,343	51.2	301	2.4	215	1.7
	Angola	20	5,198	5,112	98.4	80	1.5	6	0.1	0	0.0
	DRC	21	6,942	3,000	43.2	3,753	54.1	174	2.5	15	0.2
	Kenya	20	13,532	7,946	58.7	5,335	39.4	179	1.3	72	0.5
	Niger	11	7,070	4,734	67.0	2,335	33.0	1	0.0	0	0.0
	Nigeria	21	6,525	5,107	78.3	1,349	20.7	44	0.7	25	0.4
	Uganda	20	11,236	6,190	55.1	4,925	43.8	61	0.5	60	0.5
Asia	Cambodia	5	4,407	2,463	55.9	1,742	39.5	152	3.5	50	1.1
	China	21	13,867	8,300	59.9	4,312	31.1	511	3.7	744	5.4
	India	20	20,608	13,550	65.8	4,626	22.5	2,399	11.6	33	0.2
	Japan	10	2,972	1,809	60.9	939	31.6	197	6.6	27	0.9
	Nepal	8	7,321	6,329	86.5	907	12.4	75	1.0	10	0.1
	Philippines	17	11,907	4,754	39.9	6,380	53.6	551	4.6	222	1.9
	Sri Lanka	14	14,061	12,438	88.5	1,512	10.8	106	0.8	5	0.0
	Thailand	12	7,202	3,787	52.6	2,570	35.7	713	9.9	132	1.8
	Vietnam	15	12,202	7,798	63.9	4,072	33.4	283	2.3	49	0.4
Latin America	Argentina	14	9,720	4,153	42.7	5,028	51.7	396	4.1	143	1.5
	Brazil	19	13,437	9,373	69.8	3,724	27.7	289	2.2	51	0.4
	Cuba	17	11,383	10,153	89.2	912	8.0	284	2.5	34	0.3
	Ecuador	18	11,080	2,221	20.1	8,040	72.6	719	6.5	100	0.9
	Mexico	21	18,707	8,520	45.5	8,188	43.8	1,024	5.5	975	5.2
	Nicaragua	8	5,046	3,476	68.9	1,070	21.2	492	9.8	8	0.2
	Paraguay	6	3,091	1,531	49.5	1,482	48.0	64	2.1	14	0.5
	Peru	17	14,664	2,598	17.7	11,391	77.7	603	4.1	72	0.5
Overall		373	244,569	140,874	57.6	91,015	37.2	9,624	3.9	3,056	1.3

**Table 2:** Characteristics of the study population.

			Breastfeeding initiation			
	Total		≤1 hour		>1 hour	
Number of neonates	244,569		140,874	57.6	103,695	42.4
Maternal age (years)						
<20	30,675	12.5	17,699	12.6	12,976	12.5
20–34	187,983	76.9	109,221	77.5	78,762	76
>35	25,483	10.4	13,625	9.7	11,858	11.4
Missing	428	0.2	329	0.2	99	0.1
Marital status						
Single	31,318	12.8	17,730	12.6	13,588	13.1
Married/cohabiting	212,579	86.9	122,667	87.1	89,912	86.7
Missing	672	0.3	477	0.3	195	0.2
Education (years)						
None or primary (<6)	64,870	26.5	40,109	28.5	24,761	23.9
Secondary (7–12)	135,850	55.6	76,580	54.4	59,270	57.2
Higher ( >12)	32,593	13.3	17,713	12.5	14,880	14.3
Missing	11,256	4.6	6,472	4.6	4,784	4.6
Parity						
Nullipara	104,715	42.8	59,902	42.5	44,813	43.2
Multipara	139,153	56.9	80,483	57.1	58,670	56.6
Missing	701	0.3	489	0.4	212	0.2
Antenatal care visits						
None	9,851	4.0	5,909	4.2	3,942	3.8
1–3	58,498	23.9	34,341	24.4	24,157	23.3
>4	164,995	67.5	94,989	67.4	70,006	67.5
Missing	11,225	4.6	5,635	4.0	5,590	5.4
Maternal complications during pregnancy^†^						
Yes	67,106	27.4	33,015	23.4	34,091	32.9
No	176,958	72.4	107,731	76.5	69,227	66.8
Missing	505	0.2	128	0.1	377	0.3
Gestational age (weeks)						
<36	16,670	6.8	9,185	6.5	7,485	7.2
37–41	219,349	89.7	126,747	90	92,602	89.3
>42	5,910	2.4	3,245	2.3	2,665	2.6
Missing	2,640	1.1	1,697	1.2	943	0.9
Mode of delivery						
Vaginal delivery	191,390	78.3	122,828	87.2	68,562	66.1
Caesarean delivery	53,147	21.7	18,030	12.8	35,117	33.9
Missing	32	0.0	16	0.0	16	0.0
Facility location						
Urban	208,315	85.2	116,894	83	91,421	88.1
Peri-urban	16,152	6.6	10,860	7.7	5,292	5.1
Rural	19,439	7.9	12,834	9.1	6,605	6.4
Missing	663	0.3	286	0.2	377	0.4
Capacity						
Low	59,291	24.2	36,919	26.2	22,372	21.6
Medium	100,896	41.3	58,101	41.2	42,795	41.3
High	84,382	34.5	45,493	32.6	38,528	37.1
Ownership						
Public	202,665	82.9	121,538	86.3	81,127	78.2
Private	7,388	3	2,972	2.1	4,416	4.3
Others	30,613	12.5	14,558	10.3	16,055	15.5
Missing	3,903	1.6	1,806	1.3	2,097	2
Average number of beds in use						
<50	105,217	43	58,981	41.9	46,236	44.6
50–100	58,462	23.9	34,042	24.1	24,420	23.5
>100	78,137	32	46,647	33.1	31,490	30.4
Missing	2,753	1.1	1,204	0.9	1,549	1.5
Protocols/guidelines for postnatal and/or neonatal care						
None	33,125	13.5	16,992	12.1	16,133	15.5
Available	211,071	86.3	123,795	87.8	87,276	84.2
Missing	373	0.2	373	0.1	286	0.3
Region						
Africa	62,894	25.7	37,621	26.7	25,273	24.4
Latin America	87,128	35.6	42,025	29.8	45,103	43.5
Asia	94,547	38.7	61,228	43.5	33,319	32.1

^†^Medical conditions during pregnancy included chronic hypertension, pregnancy-induced hypertension, diabetes mellitus, cardiac and renal disorders, chronic respiratory conditions, pyelonephritis or urinary infections and other medical conditions.

**Table 3:** Odds ratios for breastfeeding initiation within 1 hour after birth.

	OR (95% CI)		AOR (95% CI)	
Maternal age (years)				
20–34	1		1	
<20	0.96	(0.81–1.12)	1.05	(0.92–1.19)
≥35	0.85	(0.73–0.96)**	0.91	(0.83–1.01)
Marital status				
Married/cohabiting	1		1	
Single	0.94	(0.74–1.19)	1.08	(0.84–1.39)
Education (years)				
None or primary (<6)	1		1	
Secondary (7–12)	0.83	(0.70–0.99)*	0.86	(0.73–1.02)
Higher (>12)	0.76	(0.59–0.97)*	0.83	(0.66–1.07)
Parity				
Multipara	1		1	
Nullipara	0.99	(0.91–1.07)	0.95	(0.88–1.04)
Antenatal care visits				
>4	1		1	
None	1.09	(0.87–1.37)	0.84	(0.67–1.04)
1–3	1.04	(0.82–1.32)	0.82	(0.62–1.01)
Maternal complications during pregnancy^†^				
No	1		1	
Yes	0.62	(0.52–0.73)***	0.76	(0.65–0.88)***
Gestational age (weeks)				
37–41	1		1	
≤36	0.87	(0.65–1.17)	0.81	(0.60–1.10)
≥42	0.87	(0.72–1.05)	0.95	(0.78–1.14)
Mode of delivery				
Vaginal delivery	1		1	
Caesarean delivery	0.29	(0.22–0.38)***	0.28	(0.22–0.37)***
Health facility location				
Urban	1		1	
Peri-urban	1.44	(0.75–2.74)	1.13	(0.57–2.24)
Rural	1.54	(0.79–2.97)	1.34	(0.60–3.00)
Health facility capacity				
Low	1		1	
Medium	0.86	(0.54–1.36)	0.97	(0.57–1.63)
High	0.80	(0.49–1.32)	0.88	(0.60–1.56)
Ownership				
Public	1		1	
Private	0.64	(0.26–1.58)	0.58	(0.21–1.62)
Others	0.62	(0.30–1.26)	0.60	(0.31–1.15)
Average number of beds in use				
<50	1			
50–100	1.06	(0.70–1.62)	1.21	(0.79–1.88)
>100	1.10	(0.67–1.78)	1.40	(0.79–2.49)
Guidelines for postnatal/neonatal care				
No	1			
Available	1.35	(0.93–2.62)	2.05	(1.07–3.92)*
Region				
Africa	1		1	1
Latin America	0.63	(0.41–0.99)*	0.85	(0.51–1.41)
Asia	1.36	(0.89–2.08)	1.54	(0.87–2.74)

(N = 213,908 deliveries at 352 health facilities). OR: odds ratio; CI: confidence interval; AOR: adjusted odds ratio. ^†^Medical conditions during pregnancy included chronic hypertension, pregnancy-induced hypertension, diabetes mellitus, cardiac and renal disorders, chronic respiratory conditions, pyelonephritis or urinary infections and other medical conditions.

*p < 0.05, **p < 0.01, ***p < 0.001.
